# Toward a Multidimensional Understanding of Misophonia Using Cluster-Based Phenotyping

**DOI:** 10.3389/fnins.2022.832516

**Published:** 2022-03-28

**Authors:** Jordan E. Norris, Suzanne H. Kimball, Danna C. Nemri, Lauren E. Ethridge

**Affiliations:** ^1^Department of Psychology, University of Oklahoma, Norman, OK, United States; ^2^Department of Communication Sciences and Disorders, University of Oklahoma Health Sciences Center, Oklahoma City, OK, United States; ^3^Section on Developmental and Behavioral Pediatrics, Department of Pediatrics, University of Oklahoma Health Sciences Center, Oklahoma City, OK, United States

**Keywords:** misophonia, cluster analysis, sensory sensitivity, auditory, phenotype

## Abstract

Misophonia is a condition characterized by hypersensitivity and strong emotional reactivity to specific auditory stimuli. Misophonia clinical presentations are relatively complex and reflect individualized experiences across clinical populations. Like some overlapping neurodevelopmental and neuropsychiatric disorders, misophonia is potentially syndromic where symptom patterns rather than any one symptom contribute to diagnosis. The current study conducted an exploratory k-means cluster analysis to evaluate symptom presentation in a non-clinical sample of young adult undergraduate students (*N* = 343). Individuals participated in a self-report spectrum characteristics survey indexing misophonia, tinnitus severity, sensory hypersensitivity, and social and psychiatric symptoms. Results supported a three-cluster solution that split participants on symptom presentation: cluster 1 presented with more severe misophonia symptoms but few overlapping formally diagnosed psychiatric co-occurring conditions; cluster 3 was characterized by a more nuanced clinical presentation of misophonia with broad-band sensory hypersensitivities, tinnitus, and increased incidence of social processing and psychiatric symptoms, and cluster 2 was relatively unaffected by misophonia or other sensitivities. Clustering results illustrate the spectrum characteristics of misophonia where symptom patterns range from more “pure” form misophonia to presentations that involve more broad-range sensory-related and psychiatric symptoms. Subgroups of individuals with misophonia may characterize differential neuropsychiatric risk patterns and stem from potentially different causative factors, highlighting the importance of exploring misophonia as a multidimensional condition of complex etiology.

## Introduction

Misophonia is a condition characterized by hypersensitivity and adverse reaction to individual-specific auditory stimuli triggering impulsive emotional reactions and autonomic arousal (Edelstein et al., 2013; Schroder et al., 2013; Cavanna, 2014). While misophonia is an auditory condition by definition, it has not yet been mapped to specific neural auditory generators. Recent work has demonstrated that misophonia triggers eliciting emotional reactions are accompanied by autonomic arousal with altered neural activity in the auditory cortex and the salience network (Schroder et al., 2019). Most individuals with misophonia have normal hearing sensitivity but exhibit increased limbic and autonomic nervous system activation suggesting that misophonia results from increased neural connections within auditory, limbic, and autonomic nervous system pathways [Wu et al., 2014; Jastreboff and Jastreboff, 2015; Brout et al., 2018; Palumbo et al., 2018; Cerliani and Rouw, 2020 (Preprint)]. More specifically, abnormal functional connectivity of anterior insular cortex (AIC) has been noted in individuals with misophonia where increased functional connectivity was specific to trigger sounds (Kumar et al., 2017). Additional increases in functional connectivity in misophonia have been noted between auditory, visual, and motor cortices, highlighting the complex nature of sensory relationships in this condition (Kumar et al., 2021). Further, individuals with misophonia exhibit reduced N1 peak averages compared to neurotypical controls suggesting underlying neurobiological differences contributing to auditory processing impairment (Schroder et al., 2014). Many of these neural findings are not unique to misophonia and show significant overlap with other disorders in which sensory processing may be impaired, such as autism spectrum disorder (ASD) and schizophrenia (Burns et al., 2017; Takarae and Sweeney, 2017; Cardon, 2018; Stefanelli et al., 2020; Koshiyama et al., 2021). Overlapping phenotypes may allow researchers to leverage the large literature arising from these disorders to better define neural targets for study, and ultimately, intervention in misophonia.

Current investigations on misophonia prevalence rates suggest that approximately 20% of the population report clinically relevant levels of misophonia symptoms (Schroder et al., 2013; Wu et al., 2014). Individuals identified as having misophonia present with anxiety, hypersensitivities to auditory stimuli, severe emotional fluctuations when exposed to specific auditory triggers accompanied by autonomic arousal, and in some cases compulsive behavior (Edelstein et al., 2013; Schroder et al., 2013; Wu et al., 2014; Rouw and Erfanian, 2018; Brout et al., 2018). Interestingly, mechanisms underlying misophonia may bear resemblance to synesthesia with emotional responsivity occurring concurrently with specific auditory sensory experiences. Initial misophonia cases were thought to constitute deviant presentations of other auditory conditions, anxiety disorders, and obsessive-compulsive disorders (OCD). However, results from a single-site study suggest symptom presentation follows a consistent pattern and exhibits a similar development path across patients lending support to the call for classification of misophonia as a discrete psychiatric disorder (Schroder et al., 2013; Jager et al., 2020).

Systematic investigation of misophonia is a relatively new field, primarily focused on characterizing misophonia, formally defining diagnostic criteria, and developing tools to identify misophonia. A pressing goal is creating the space *via* universal language specific to misophonia necessary to develop effective treatments and support for misophonia based on evaluations of misophonia features. Recent efforts provided a common understanding of misophonia and created a consensus definition of misophonia based on expert evaluation of existing published literature that defined misophonia as a disorder [Swedo et al., 2021 (Preprint)]. Most studies assessing misophonia utilized questionnaires to informally diagnose misophonia, with only three known studies performing full medical and psychiatric evaluation of participants (Schroder et al., 2013; Erfanian et al., 2019; Jager et al., 2020). More recent studies investigating misophonia and sound hypersensitivity have also used psychoacoustic methods, providing a quick and reliable means of assessing misophonia (Dozier and Morrison, 2017; Enzler et al., 2021; Hansen et al., 2021). However, limited studies have explored variability in the clinical presentation of misophonia (Cassiello-Robbins et al., 2021; Hansen et al., 2021). Like some of the overlapping neurodevelopmental and neuropsychiatric disorders, misophonia may actually reflect a syndrome, in which any given symptom may be present or absent, but the constellation of symptoms produces the diagnosis. Understanding clinical symptoms and features of misophonia is a key area of research that remains underexplored and is necessary to confirm its clinical nature (Brout et al., 2018). Clustering techniques following the research domain criteria approach (RDoC – a research framework designed to integrate various levels of information and approaches to assessing and understanding neuropsychiatric conditions with the goal of improving diagnostic and treatment/support service options) have been useful in characterizing subgroups of individuals in syndromic conditions who share common features and thus may have similar underlying biology (Clementz et al., 2016). Identification of subpopulations based on symptom clustering is a novel approach to identifying underlying pathophysiology in misophonia.

### Symptom Presentation

Auditory hypersensitivity and behavioral responses with misophonia are typically evoked by specific patterns of auditory stimuli, referred to as triggers (Erfanian et al., 2019). Auditory triggers vary across individuals and consist of common sounds from organic sources including eating, breathing, certain speech sounds, and other non-organic or environmental sounds (Erfanian et al., 2019; Jager et al., 2020). Extreme sensitivity and emotional responses to auditory stimuli negatively impact quality of life making it difficult for individuals with misophonia to engage in situations or environments that expose them to auditory triggers. When exposed to common triggers, the severity level of misophonia symptoms is associated with decreased cognitive control (Daniels et al., 2020). Specifically, individuals with misophonia show increased difficulties with selective attention tasks when distracted by their trigger sounds, and evidence suggests the additional presence of tinnitus in some individuals may exacerbate this response (Silva and Sanchez, 2019; Frank et al., 2020).

Misophonia is a complex condition that commonly presents with co-occurring symptoms across allied health disciplines (Erfanian et al., 2019). Sensory hypersensitivity symptoms and co-occurrence of tinnitus and hyperacusis with misophonia are particularly of interest because the presence of conditions with auditory parameters implies the possibility that basic sensory processing is broadly affected in those with misophonia (Sztuka et al., 2010).

### Sensory Processing Disorders

Investigations aiming to define diagnostic criteria for misophonia and evaluate symptom presentation have widely focused on behavioral and emotional components of misophonia with limited efforts to explore sensory processing aspects of misophonia (Jager et al., 2020). Recent work suggests potential associations between misophonia and anxiety disorders, as well as sensory processing disorder (SPD), ASD, and tinnitus (Schroder et al., 2013; Cassiello-Robbins et al., 2021). Auditory sensory processing abnormalities are both commonly present in misophonia including general sensory processing differences and sensory hypersensitivity (Wu et al., 2014). ASD is another condition associated with anxiety and central processing abnormalities. Autistic individuals commonly present with auditory hypersensitivities that are either specific or non-specific to auditory triggers. It is possible that individuals with misophonia experience subthreshold ASD-like sensory symptoms reflecting the broad autism phenotype (BAP) (Hurley et al., 2007).

### Tinnitus

Tinnitus is a complex phenomenon stemming from a basic auditory sensory processing abnormality with variable pathogenesis. Recent efforts proposed a new definition where tinnitus is defined as an auditory sensation without an external sound stimulation that potentially impacts quality of life as a lived unpleasant experience. Two types of tinnitus exist: objective and subjective. Objective tinnitus is caused by an internally generated stimulus (i.e., stemming from physiological fluctuations typically in the auditory pathway) and subjective tinnitus is idiopathic (i.e., has no identifiable acoustic source) (Noreña et al., 2021). Others have proposed tinnitus as a discrete psychiatric condition (i.e., tinnitus disorder) when associated with emotional distress and separate from tinnitus experiences without suffering (De Ridder et al., 2021). It is estimated that misophonia occurs in approximately 10–60% of individuals that experience any form of tinnitus (Sztuka et al., 2010; Wu et al., 2014). Individuals that present with clinically relevant levels of misophonia symptoms exhibit general sensory over-responsivity indicating a likelihood that basic sensory processing abnormalities (e.g., tinnitus, hyperacusis, etc.) contribute to increased likelihood of misophonia (Sztuka et al., 2010; Jastreboff and Jastreboff, 2015). Whether tinnitus leads to misophonia, represents a symptom of misophonia, or remains a common co-occurring condition remains unclear; however, tinnitus pathology has been consistently linked with neuroplastic changes within the central auditory pathway between the cortex and the cochlea, areas which have been proposed to be affected in misophonia (Henry et al., 2014). Misophonia and tinnitus both may occur with emotional distress increasing diagnostic difficulties when patients present with both and highlights a need to understand the rate of co-occurrence and underlying physiological mechanisms of overlap (De Ridder et al., 2021). Indeed, misophonia, ASD, tinnitus, and hyperacusis may share some pathological mechanisms contributing to the sensory processing aspects of these conditions (Jastreboff and Jastreboff, 2015). Auditory sensation and perceptual conditions like tinnitus and hyperacusis are more prevalent among populations of individuals with sensory processing disorders (i.e., ASD). Rates of tinnitus in populations with ASD are similar to high rates of tinnitus seen in populations with misophonia (Danesh et al., 2015).

### Current Study

The link between misophonia and other sensory processing disorders remains to be fully understood. Given the overlap in symptoms with a number of syndromic conditions, suggesting both basic sensory and neuropsychiatric (e.g., anxiety) involvement, and the response range to trigger sounds noted in individuals with misophonia, characterization of symptom clusters may be beneficial in understanding underlying pathophysiology and variability in misophonia. Current efforts aim to explore the possibility that sensory processing is broadly affected in misophonia, and that symptom clusters can be used to better define sub-populations in misophonia. Using clustering-based methods to categorize a population of college students, based on previous reports (Schroder et al., 2013; Wu et al., 2014) we expect to find approximately 20% reporting clinically relevant levels of misophonia symptoms, and that clusters most representative of individuals with high misophonia symptoms will also show increased prevalence of other co-occurring conditions such as tinnitus and sensory processing disorders, as well as increased prevalence of broad autism phenotype characteristics.

## Materials and Methods

### Participants

Participants were undergraduate students (*N* = 343) at the University of Oklahoma (OU) in Norman, Oklahoma. Participants were predominately female [69.7%; consistent with other studies where participants opt into participation; (Wu et al., 2014; Dozier and Morrison, 2017)] and ranged from ages 18 to 36 (*M* = 18.96, *SD* = 1.7) (Table 1) with a primary vocation of student (*N* = 331; 96.5%). Of 343 participants, 263 were Caucasian/White (76.7%), 32 were Black/African American (9.3%), 46 were Latino/Hispanic (13.4%), 28 were Asian/Asian American (8.2%), 26 were American Indian/Alaska Native (7.6%), 2 were Hawaiian/Other Pacific Islander (0.6%), and 2 identified as other (0.6%). Self-report current diagnoses were also collected.

**TABLE 1:** Demographics.

Variable	
Age (Years)	*M* = 18.96 *SD* = 1.7
**Gender**	
Male	104 Male (30.3%)
Female	239 Female (69.7%)
Non-binary	0 Non-binary (0%)
**Ethnicity**	
Caucasian	*N* = 263 (76.7%)
Black/African American	*N* = 32 (9.3%)
Latino/Hispanic	*N* = 46 (13.4%)
Asian/Asian American	*N* = 28 (8.2%)
American Indian/Alaska Native	*N* = 26 (7.6%)
Native Hawaiian/Other Pacific Islander	*N* = 2 (0.6%)
Other	*N* = 2 (0.6%)
**Education**	
Less than high school degree	*N* = 2 (0.6%)
High school graduate	*N* = 129 (37.6%)
Some years of college/university (no degree)	*N* = 194 (56.6%)
Vocational training	*N* = 2 (0.6%)
Associates degree	*N* = 8 (2.3%)
Bachelor’s degree	*N* = 5 (1.5%)
Master’s degree	*N* = 1 (0.3%)
Professional degree	*N* = 0 (0%)
Doctorate degree	*N* = 0 (0%)

Participants were recruited using a secure online research participation system through the university’s undergraduate psychology research participation pool and all data were collected anonymously *via* Qualtrics™. Those who completed the survey received 1 h worth of class credit. All study procedures were approved by the OU Institutional Review Board (IRB). All participants electronically acknowledged their informed consent to participate prior to completing the survey. All responses remained anonymous and no personal identifiable information was collected from participants.

### Measures

The final survey was designed to address an array of symptoms characteristic of or that may overlap with misophonia, referred to as the Spectrum Characteristic Survey (SCS). The SCS was comprised of a demographics section and six clinical measures designed to address various aspects of misophonia, related symptoms, and co-occurring conditions.

#### Misophonia Questionnaire

The Misophonia Questionnaire (MQ) is a validated, three-part, 20-item self-report measure designed to index misophonia symptoms (Wu et al., 2014). Part one assessed specific auditory triggers associated with misophonia, part two evaluates ensuing emotions and behaviors associated with misophonia-related triggers, and part three measures sound sensitivity severity. Participants were asked to rate their sensitivity to auditory triggers on a scale from 0 (“not at all true”) to 4 (“always true”). The MQ was utilized to assess the potential presence of misophonia, gauge trigger responses, and symptom severity. MQ severity was utilized to split participants into groups reflecting clinically or non-clinically relevant levels of misophonia symptoms based on threshold scores of 7 out of 10 (Wu et al., 2014). Participants were additionally asked if they had any triggers in other sensory domains, to assess presence of triggers in other sensory modalities. Wu et al. (2014) reported high internal consistencies for total scores (α = 0.89), and both subscales (emotions and behaviors: α = 0.89, symptom scale α = 0.86). Further, the MQ also demonstrated high convergent and discriminate validity indicating that the MQ significantly discriminated misophonia from other types of sensory defensiveness. Results were replicated in Zhou et al. (2017). Although not the only measure available for assessing misophonia symptoms, the MQ is one of the more commonly used and thus allows better generalization of results (Brout et al., 2018; Potgieter et al., 2019).

#### S-Five (2018)

The S-Five is a self-report psychometric tool for evaluating misophonia presence and related symptoms [Vitoratou, 2018 (Preprint)]. The initial version of the S-Five published in 2018 was utilized for the purposes of the current study. The S-Five is a 98-item measure that assessed two aspects of misophonia: (1) triggers and (2) statements regarding behavior associated with misophonic triggers. The S-Five was used to further evaluate the triggers and trigger responses (i.e., behaviors associated with sensory sensitivity to sound triggers). Participants were asked to rate their typical reaction to trigger items on a scale from 0 (“does not bother me”) to 5 (“so unbearable that I need to plan beforehand to avoid it”). The version used in the current study was the 2018 version of the S-Five and was used for broad investigation of misophonia, but was not the validated version available after completion of data collection for the current study (Vitoratou et al., 2021).

#### Tinnitus Handicap Inventory

Participants who responded affirmatively to the screening question, “Do you experience tinnitus (ringing in the ears)?” received the Tinnitus Handicap Inventory (THI). The THI is a self-report 25-item measure designed to identify, quantify, and evaluate tinnitus severity as well as tinnitus’ impact on participant quality of life. The THI is a valid and reliable measure of tinnitus-related difficulties in individuals that report experiencing tinnitus demonstrating both convergent and construct validity (Newman et al., 1996). THI has functional limitations and should be interpreted as an index of tinnitus impact on quality of life (Meikle et al., 2012).

#### Adolescent/Adult Sensory Profile

The Adolescent and Adult Sensory Profile (ASP) is a reliable and valid self-report 6-part measure of sensory processing patterns and effects on function performance. Scoring assessed only the auditory processing block and four sensory behavior quadrants: low registration, sensation seeking, sensory sensitivity, and sensation avoiding. The ASP specifically indexed individual responses to sensations, as opposed to an individual’s general response or cognitive appraisal of a stimulus. On validation, the ASP demonstrated good reliability, internal consistency, and construct validity (Brown et al., 2001).

#### Broad Autism Phenotype Questionnaire

The Broad Autism Phenotype Questionnaire (BAPQ) assesses a set of characteristics that encompass personality and language traits reflecting phenotypical expression of the genetic predisposition for ASD. The BAP term is typically applied to those who exhibit mild personality and cognitive traits observed in autistic individuals (Hurley et al., 2007; Landry and Chouinard, 2016). The use of the BAPQ addressed a potential relationship between BAP and misophonia within a general population of young adults. The BAPQ is reliable and demonstrated good internal consistency and construct validity (Hurley et al., 2007).

#### Schizotypal Personality Questionnaire – Brief Revised (Updated)

The Schizotypal Personality Questionnaire – Brief Revised (Updated) (SPQ-BRU) is a self-report evaluation of schizotypy and vulnerabilities to certain features of neurodevelopmental and schizophrenia spectrum disorders. The revised SPQ demonstrated reliability and both convergent and discriminant validity (Davidson et al., 2016). This measure was specifically chosen to evaluate broad neuropsychiatric risk as it relates to prevalence rates of broad autism phenotype characteristics and sensory processing disorders.

### Data Analysis

All statistical analyses were conducted using IBM SPSS Statistics 27 (IBM Corp, 2020). Descriptive statistics were calculated for demographic variables, tinnitus presence and severity, sensory triggers, and misophonia presence. Independent samples *t*-tests were conducted to evaluate potential differences in variable scores between individuals with clinically relevant levels of misophonia and individuals without significant misophonia symptoms as an exploratory and descriptive endeavor. Evaluation of potential sex effects was conducted through multivariate analyses of variance (ANOVA) based on reports of phenotypical differences between males and females with neurodevelopmental disorders sharing symptom characteristics (Ethridge et al., 2017, 2019; May et al., 2019). Age was included as a covariate and retained when significant to control for age-related factors that potentially influence symptom experiences, presentation, and quality of life (Schroder et al., 2013; Palumbo et al., 2018; Jager et al., 2020).

#### Cluster Analyses

Scored variables from clinical measures (*N* = 16, see Table 2 for a full list of included measures) were standardized using *z*-scores for cluster analyses. Variables were selected for clustering based on hypothesized relationships to misophonia or psychiatric risk. Subscales were selected in lieu of total scores to avoid issues with interpreting outcomes associated with combining subscales measuring different symptoms thus preventing a less accurate assessment of sub-phenotypes (e.g., MQ: used the subscale for emotional behaviors and trigger responses over total score). Subgroup formation was determined with the use of Two-Step cluster analysis and silhouette plot evaluation as a data-driven approach to determining the initial input for k-means clustering. The Two-Step cluster analysis outcome was confirmed using silhouette plot evaluation, as the results of silhouette plotting are representations of clustering method outputs. A Two-Step cluster approach identifies sub-groups by running pre-clustering followed by hierarchical clustering methods and provides an estimation for the optimal cluster definition. The Two-Step cluster algorithm outcome suggested two subgroups splitting on the presence or absence of clinically significant levels of misophonia symptoms, however silhouette plots suggested the presence of a third subgroup. Due to this discrepancy, we conducted a two-cluster solution and a three-cluster solution *via* K-Means Cluster analyses to explore and address potential splits on variable types. K-means clustering provides cluster centroids based on minimizing the sum of squared simple Euclidian distance for the pre-defined cluster number. The k-means algorithm achieved stability after 26-iterations for the three-cluster solution and after 5-iterations for the two-cluster solution. Univariate ANOVAs were run to address group differences by cluster on variables entered into the k-means cluster analysis according to the three-cluster solution. Fisher’s Least Significant Difference (Fisher’s LSD) *post-hoc* test determined significance between clusters. Current diagnoses were also evaluated by cluster membership according to the three-cluster solution using chi-square analyses.

**TABLE 2:** Results of independent samples *t*-tests comparing misophonia groups on scored clinical variables.

	Misophonia		No misophonia				
		
Clinical variables	M	SD	M	SD	*t*	*df*	Cohen’s *d*
MQ sounds sensitivity	16.30	5.00	11.36	4.88	−6.75***	336	–1.01
MQ emotional behaviors	21.81	6.89	12.51	6.41	–9.49***	331	–1.43
MQ total	38.10	9.12	23.97	9.55	–9.86***	328	–2.93
THI total	24.00	16.55	14.66	16.48	–3.04**	165	–0.57
ASP – Auditory processing	31.69	6.84	27.08	6.60	–4.67***	337	–0.69
ASP – Low registration	40.00	8.05	34.94	9.193	–3.78***	333	–0.56
ASP – Sensory seeking	47.15	8.84	46.53	8.48	–0.47	332	–0.07
ASP – Sensory sensitivity	43.94	9.67	37.98	9.18	–4.29***	330	–0.64
ASP – Sensation avoiding	41.27	8.43	37.41	8.97	–2.87**	329	–0.43
BAPQ total	3.28	0.43	3.02	0.55	–3.78***	84.11	–0.48
BAPQ – Aloof	3.09	0.70	2.94	0.85	–1.32	83.49	–0.17
BAPQ – Pragmatic language	2.95	0.47	2.67	0.55	–3.41***	335	–0.51
BAPQ – Rigid	3.54	0.56	3.19	0.62	–3.75***	335	–0.56
SPQ – Cognitive perceptual	39.76	7.8	34.22	8.9	–4.26***	335	–0.63
SPQ – Interpersonal	31.15	7.61	27.52	8.53	–2.89**	337	–0.43
SPQ – Disorganized	24.56	6.09	22.49	6.32	–2.21*	333	–0.33
S-Five triggers 1	23.98	9.88	14.44	7.67	–6.54***	61.49	–1.19
S-Five triggers 2	19.88	12.47	10.59	8.30	–5.17***	59.73	–1.02
S-Five triggers 3	12.45	8.57	7.76	6.01	–3.82***	61.97	–0.75
S-Five presence	48.24	12.88	34.32	11.27	–8.14***	340	–1.21
S-Five emotional experience	61.38	16.10	44.85	13.22	–8.05***	334	–1.07
S-Five reaction behaviors	60.20	15.49	45.16	13.81	–7.19***	338	–1.07
S-Five perceptions of misophonia	27.15	10.79	18.44	9.19	–6.65***	330	–0.92

Mean values for all clinical variables by group and t-scores. T-scores accompanied by non-whole number degrees of freedom are t-tests without assumed variance. p > 0.05, *p < 0.05, **p < 0.01, ***p < 0.001.

#### Mediation Model

To investigate the role of anxiety on the relationship between misophonia symptoms severity and emotional behaviors measured *via* the MQ a bootstrapped simple mediation analysis was performed using PROCESS (Hayes, 2018). Simple self-reported anxiety frequency and intensity were used as individual mediation variables. Anxiety was assessed by asking participants the frequency of which anxiety was experienced (5-point scale from *never* – *all the time*) and the intensity of anxiety experienced in a typical day (5-point scale from *none* – *extreme distress*). Further bootstrapped mediation analyses were conducted to evaluate anxiety intensity and frequency mediation by cluster. All mediation analysis were bootstrapped 5,000 times.

## Results

Of 343 participants, 54 (15.6%) reported clinically relevant levels of misophonia symptoms indicated by self-reported scores of 7 or greater on the MQ Misophonia Severity Scale. All scored clinical measure variables from the SCS were assessed by group (above and below threshold for clinically significant misophonia) for differences with resulting significance for variables indexing sensory sensitivity. Significant group differences were identified for 14 out of 16 scored clinical variables that addressed various symptoms of misophonia with the misophonia group exhibiting increased scores compared to the below threshold (non-misophonia) group (Table 2).

Additional intrapersonal variables that potentially interact with misophonia presence were explored *via* three-way MANCOVA on questionnaire variables. All questionnaire variables were assessed by sex and misophonia diagnosis (group) controlling for age. Only one variable exhibited significant sex differences for individual misophonia symptoms endorsed across the whole sample, suggesting misophonia affects males and females similarly. Only the aloof subscale scores of the BAPQ significantly differed by sex, *F*_(1,129)_ = 4.23, *p* = 0.042, but the lack of interaction between misophonia and sex for the BAPQ aloof subscale suggests this sex difference is not linked to misophonia. The only significant interaction was found between group and sex for total THI score, *F*_(1,129)_ = 10.94, *p* = 0.001. Females with clinical levels of misophonia symptoms reported greater tinnitus symptom severity (*M* = 27.82, *SD* = 14.05) compared to males with clinical levels of misophonia symptoms (*M* = 10.00, *SD* = 7.35) and females and males without misophonia based on MQ severity scores (females: *M* = 11.45, *SD* = 10.53; males: *M* = 18.38, *SD* = 20.22).

### Trigger Endorsement

Mean, standard deviation, item ranges, and frequency of endorsement for S-Five Misophonia triggers are presented in Table 3 and for the MQ triggers in Table 4. S-Five trigger items were evaluated by misophonia grouping. Individuals that qualified for misophonia reported significantly decreased auditory stimulus tolerance for the specific auditory triggers listed in Table 3 compared to those that did not qualify for misophonia. The S-Five also included non-auditory triggers that were significantly different between those who qualified for misophonia and those that did not. Trigger items from the MQ were also evaluated by misophonia grouping. Individuals who qualified for misophonia reported significantly increased auditory trigger sensitivity to all trigger items on the MQ compared to participants who did not qualify for misophonia (Table 4).

**TABLE 3:** Trigger items endorsed across all participants on the trigger section of the S-Five.

				Frequency of endorsement					
				
S-Five trigger item	*t*	M	SD	0	1	2	3	4	5
Loud chewing	–4.64***	2.09	1.09	25	67	140	77	27	6
Crunching an apple	–5.25***	0.91	1.17	179	72	49	30	10	2
Swallowing	–4.59***	0.92	1.14	163	96	45	22	13	2
Lip smacking	–5.24***	1.85	1.23	51	88	106	60	32	5
Slurping	–3.74***	1.64	1.27	77	88	94	50	28	4
Breathing	–4.27***	0.75	1.03	186	89	42	16	5	3
Throat clearing	–5.34***	1.01	1.06	139	105	59	34	4	1
Coughing	–3.41***	0.97	1.06	148	96	64	28	5	1
Nose sniffing	–4.92***	1.09	1.08	123	110	70	27	9	1
Baby crying	–0.32	1.78	1.21	59	81	103	77	13	7
Repetitive barking	–2.46*	1.95	1.18	36	96	96	75	33	3
Certain letter sounds	–2.32*	0.32	0.70	268	44	22	6	1	0
Certain accents	–1.56	0.30	0.74	280	34	18	7	3	0
Hiccups	–2.74**	0.59	0.83	195	106	28	9	2	1
Tapping pen	–3.29**	1.45	1.14	73	127	79	45	15	3
Tapping foot	–4.71***	1.18	1.12	111	117	72	29	12	2
Tapping finger	–4.21***	1.14	1.13	121	109	63	36	9	2
Swinging legs	–3.85***	0.63	0.97	213	72	38	14	5	1
Clicking pen	–3.94***	1.59	1.16	64	109	95	54	15	4
Keyboard tapping	–3.54***	0.76	1.05	192	74	43	26	4	1
Rustling plastic	–3.83***	1.06	1.05	126	109	73	25	7	1
Whistling sound	–3.21**	0.96	1.09	153	99	53	27	11	0
Rustling paper	–3.39**	0.81	1.00	173	91	57	13	8	1
Car engine	–1.85	0.41	0.80	254	52	23	13	1	0
Clock ticking	–4.78***	0.91	1.15	167	92	49	19	11	4
Humming of object	–4.18***	0.89	1.11	169	93	43	28	8	2
Low frequency bass sounds	–3.11***	0.56	0.96	229	63	31	13	6	1
Skin picking	–1.06	1.10	1.27	155	77	55	37	15	4
Foot wiggling	–2.68**	0.51	0.90	235	61	28	13	3	1
Hair twirling	–1.77	0.32	0.74	276	33	23	9	1	0
Pacing	–2.83**	0.81	1.04	173	97	44	16	11	0
Nail biting	–2.08*	0.78	1.07	187	82	46	18	6	3
Hands to mouth	–1.77	0.60	0.94	214	77	33	12	7	0
Slimy textures	–2.16*	1.35	1.31	114	89	74	42	12	10
Strong smells	–2.62*	1.95	1.29	59	65	90	100	17	11
Seeing someone chew gum	–3.84***	0.65	1.12	230	51	32	17	9	4

All items are on a scale of 0–5 and not all triggers are auditory-specific stimuli. Trigger items are from the 2018 version of the S-Five psychometric tool for Misophonia evaluation. T-scores reflect independent samples Welch’s t-tests comparing trigger item endorsement by group (misophonia vs no misophonia). p > 0.05, *p = 0.05, **p = 0.01, ***p = 0.001.

**TABLE 4:** Trigger items endorsed on the trigger subscale of the misophonia questionnaire (MQ).

				Frequency of endorsement				
				
MQ trigger item	*t*	M	SD	0	1	2	3	4
People eating	–4.59***	2.34	1.16	24	56	107	93	63
Repetitive tapping	–2.96**	2.06	1.19	35	86	86	94	42
Rustling	–4.69***	1.66	1.19	62	109	82	63	26
Nasal sounds	–4.21***	1.98	1.17	32	100	92	78	39
Throat sounds	–3.98***	1.91	1.21	47	89	88	83	35
Vowel/consonant sounds	–3.85***	0.79	1.01	174	102	35	25	6
Environmental sounds	–4.14***	1.39	1.19	93	109	75	44	22

All items are on a scale of 0–4. T-scores reflect independent samples t-tests comparing trigger item endorsement by group (misophonia vs no misophonia). p > 0.05, **p = 0.01, ***p = 0.001.

### Tinnitus

A total of 50.4% (*N* = 173) of participants reported experiencing ringing in the ears or tinnitus *via* the screener question across the whole sample. It is likely this includes many false positives who only experience transient ear ringing, a normal phenomenon, versus actual tinnitus, therefore percentages should be interpreted broadly as sensitivity to aural phenomena, with above-threshold THI scores reflecting more likely cases of true tinnitus. Forty participants who qualified for misophonia reported experiencing tinnitus making the rate of tinnitus or ear ringing occurrence 74.1% among the participants who qualified for misophonia. Among individuals without misophonia, 46% reported ringing in the ears *via*the screener, suggesting a marginal, χ^2^(1, 343) = 3.84, *p* = 0.05 increase in tinnitus, or at the least sensitivity to aural phenomena, in misophonia. The total incidence rate of co-occurrence of misophonia and tinnitus among all of those who reported tinnitus *via*screener was 23.1%, which is significantly higher than the overall incidence rate of misophonia in this sample (15.6%), χ^2^(1, 516) = 4.35, *p* = 0.037. THI scores provide a more informative index of true tinnitus. Of the 40 participants with co-occurring tinnitus reports *via*screener and misophonia, 37 completed the THI and reported experiencing significantly increased tinnitus severity (*M* = 24.00, SD = 16.55) compared to participants who reported without misophonia (*M* = 14.66, SD = 16.55), *t*(165) = −3.04, *p* = 0.003.

### Cluster Results

**FIGURE 1:**
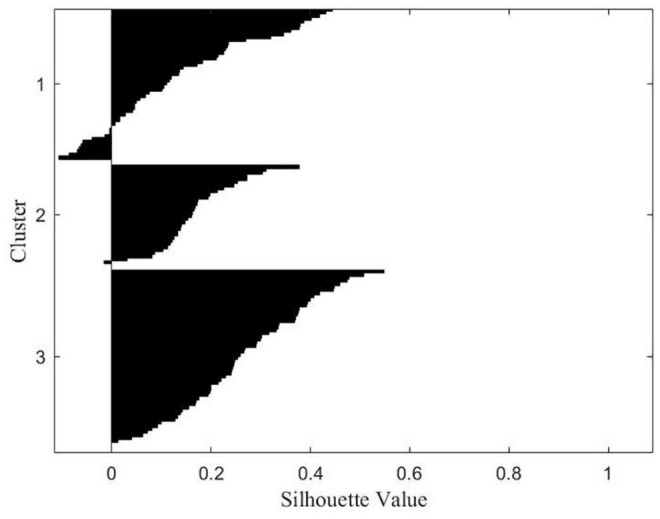
Silhouette plot for the three-cluster solution.

Multivariate taxometric analyses (i.e., clustering) did not neatly identify subcategories that well-characterized the data according to the silhouette measure of cohesion and separation. Two-Step Cluster analysis determined two distinct subgroupings with fair cluster quality based on 16 *z*-scored variable inputs where the average silhouette measure of cohesion and separation was 0.3 (i.e., potentially more variation within clusters or clusters are more similar than preferred). However, silhouette plots showed clear separation for both the two- and three-cluster solution (average silhouette scores: two-cluster solution = 0.34, three-cluster solution = 0.23) (Figure 1). To further evaluate the appropriateness of the three cluster solution, a univariate analysis of variance (ANOVA) tested for group differences on standardized scored variables by cluster membership according to a three-cluster solution. Exploratory cluster efforts found a subset of variables (*N* = 13) neatly spanning three cluster categories including variables directly indexing misophonia, tinnitus severity, sensory hypersensitivity, and social symptoms. As the majority of variables supported a three-cluster solution *via*significant differences across all three clusters, and this solution represented potential knowledge gained on statistically-supported subgroupings, the three-cluster solution was retained. Table 5 shows the breakdown of significance by variables significantly different across all three clusters (trichotomous) and those significantly different between only two clusters (dichotomous). Significant trichotomous variables from the three-cluster k-means solution imply that certain traits of misophonia evaluated in the current study exist on a spectrum. The remaining variables (*N* = 3) significantly fell into two independent clusters signifying potential threshold behavior for variables specifically indexing sensory responsivity and sensory hyposensitivity.

**TABLE 5:** Clustering behavior and ANOVA results for variables entered into the K-means cluster analysis based on the three-cluster solution.

			Dichotomous outcomes						Trichotomous outcomes					
				
Clinical variables	ANOVA		Cluster 1		Cluster 2		Cluster 3		Cluster 1		Cluster 2		Cluster 3	
							
	*F*	df	M	SD	M	SD	M	SD	M	SD	M	SD	M	SD
MQ sound sensitivity	42.67***	2, 335							0.65	0.96	–0.48	0.79	0.03	0.95
MQ emotional behaviors	55.19***	2, 330							0.68	0.93	–0.56	0.77	0.09	0.93
MQ severity	48.12***	2, 240							0.64	1.10	–0.53	0.65	0.09	0.92
THI total	22.11***	2, 164							0.01	0.82	–0.58	0.32	0.54	1.25
ASP sensory seeking	6.88***	2, 331	0.32	0.93	–0.06	1.09	–0.18	0.89						
ASP auditory processing	96.26***	2, 336							0.34	0.75	–0.75	0.87	0.58	0.75
ASP low registration	84.03***	2, 332							0.26	0.74	–0.71	0.83	0.59	0.86
ASP sensory sensitivity	100.34***	2, 329	0.42	0.76	–0.76	0.77	0.55	0.84						
ASP sensation avoiding	127.64***	2, 328							0.19	0.73	–0.77	0.71	0.76	0.81
BAPQ aloof	86.35***	2, 335	–0.30	0.77	–0.51	0.95	0.79	0.67						
BAPQ pragmatic language	99.33***	2, 334							–0.07	0.69	–0.66	0.83	0.77	0.82
BAPQ rigid	79.31***	2, 334							0.25	0.86	–0.70	0.83	0.58	0.79
BAPQ total	191.38***	2, 324							–0.09	0.64	–0.79	0.81	0.93	0.54
SPQ disorganized	49.01***	2, 332							0.07	0.95	–0.55	0.92	0.56	0.77
SPQ cognitive perceptual	68.81***	2, 334							0.08	0.86	–0.62	0.83	0.64	0.85
SPQ interpersonal	90.14***	2, 336							–0.19	0.83	–0.57	0.89	0.79	0.69

F-scores reflect ANOVA results for the three-cluster solution split by within cluster significance into dichotomous or trichotomous. All variables were z-score transformed. p > 0.05, ***p = 0.001.

The three-cluster solution grouped 93 participants into cluster 1, 132 participants into cluster 2, and 118 participants into cluster 3. Cluster 3 was significantly different from clusters 1 and 2 on all variables directly indexing misophonia and tinnitus severity (Table 5). The three-cluster solution grouped 22 participants who qualified for misophonia into cluster 3 and 31 participants who qualified for misophonia into cluster 1. Only 1 participant who exhibited clinically significant levels of misophonia clustered into cluster 2, suggesting that individuals who qualify for a misophonia diagnosis potentially exhibited one of two presentations of misophonia symptoms or co-morbidities. When applying a three-cluster solution after evaluating cluster membership for the two-cluster solution, the third cluster was predominately comprised of individuals that were previously clustered into cluster 2 from the two-cluster solution. Only three participants from cluster 1 were newly clustered into cluster 3, suggesting that the two-cluster solution primarily separated groups based on the intensity of symptoms (i.e., high or low misophonia symptoms), and the three-cluster solution further subdivides the high misophonia symptom group.

Participants were asked to self-report whether they experienced tinnitus to address the presence of basic sensory processing abnormalities. Chi-squared analysis on self-reported tinnitus presence by cluster showed significantly increased frequencies of individuals with self-reported tinnitus relative to those without tinnitus in cluster 1, χ^2^ (2, *N* = 343) = 10.16, *p* = 0.006. Cluster 2 had more participants without tinnitus than with tinnitus and cluster 3 had approximately even numbers of participants with and without tinnitus. A chi-squared analysis was also conducted for THI total scores and showed an even distribution of responses across the three clusters. However, individuals in cluster 3 appeared to endorse increased tinnitus severity (i.e., higher THI total scores) more frequently than participants in clusters 1 and 2. Chi-squared results paired with significantly increased average THI scores in cluster 3 suggest increased basic sensory processing challenges may be characteristic of cluster 3 (Table 5). Cluster 3 was also associated with significantly higher sensory symptoms on the trichotomous ASP variables, further supporting a broad sensory component for this subgroup, however similarly increased BAPQ and SPQ scores in this cluster suggest that individuals in this subgroup are more broadly affected by subclinical psychiatric symptoms in general, whereas increased MQ scores in cluster 1 coupled with more intermediate psychiatric scores may indicate a more “pure” form of misophonia.

### Other Sensory Triggers

Means and standard deviations are reported in Table 6 for self-reported triggers and the severity of trigger experience in other sensory modalities. No significant differences were reported by misophonia grouping for other sensory triggers, but severity of other sensory trigger experiences were significantly different for all sensory modalities between those that qualified for misophonia and those that did not (Table 6). Triggers in other sensory modalities were further evaluated by cluster membership using a independent samples Kruskal_Wallis test resulting in a significant main effect of cluster (Table 7). Primary differences in other sensory triggers and severity were found between cluster 1 and cluster 2 with cluster 1 exhibiting the greatest trigger endorsement and higher severity scores. Cluster 1 presenting with the greatest scores on all other sensory triggers and cluster 3 reflecting the more intermediate phenotype suggests that sensory difficulties in other modalities may be a more universal experience for those reporting clinically relevant levels of misophonia symptoms.

**TABLE 6:** Results of independent samples *t*-tests comparing misophonia groups on other sensory triggers.

	Misophonia		No misophonia			
		
Other sensory variables	*N*	Percentage	*N*	Percentage	Chi-squared	df
**Triggers**						
Visual	21	38.9%	92	31.8%	0.86	1
Smell	27	50.0%	143	49.5%	0.03	1
Taste	19	35.2%	93	32.2%	0.23	1
Texture	32	59.3%	127	43.9%	3.34	1

	**Misophonia**		**No misophonia**			
		
	**M**	**SD**	**M**	**SD**	***Z***	***U***

**Severity**						
Visual	1.43	1.25	0.91	1.15	–3.24**	5104
Smell	1.88	1.52	1.32	1.27	–2.57*	5559
Taste	1.71	1.50	0.96	1.17	–3.48**	4843.5
Texture	2.13	1.66	1.24	1.30	–3.71***	5007

Report of other sensory triggers by sensory modality, including percentage of total group (misophonia vs no misophonia). Mean values for level of other sensory trigger severity by group and z-scores. Z-scores reflect Mann-Whitney tests. p > 0.05, *p < 0.05, **p < 0.01, ***p < 0.001.

**TABLE 7:** Clustering behavior and kruskal-wallis test results for other sensory triggers based on the three-cluster solution.

	Chi-squared		Cluster 1		Cluster 2		Cluster 3	
				
Other sensory variables	*X* ^2^	df	*N*	%	*N*	%	*N*	%
**Triggers**								
Visual	8.88**	2	41	46.1%	33	26.4%	39	35.1%
Smell	6.83*	2	56	60.2%	55	42.6%	59	52.2%
Taste	14.70**	2	38	41.3%	27	21.6%	47	43.1%
Texture	21.05***	2	56	60.9%	40	32.0%	63	54.8%

	**Kuskal-Wallis**		**Cluster 1**		**Cluster 2**		**Cluster 3**	
				
	***H***	**df**	**M**	**SD**	**M**	**SD**	**M**	**SD**

**Severity**								
Visual	13.02**	2	1.29	1.29	0.66	0.99	1.01	1.12
Smell	16.04***	2	1.73	1.47	0.97	1.12	1.44	1.35
Taste	16.49***	2	1.38	1.44	0.67	0.98	1.24	1.28
Texture	28.72***	2	1.84	1.59	0.77	1.05	1.52	1.39

Chi-squared results for endorsement of triggers in other sensory modalities, with number and percentage of each cluster. Group differences on trigger severity across clusters assessed using Kruskal-Wallis, with H scores reported. p > 0.05, *p = 0.05, **p = 0.01, ***p = 0.001.

### Anxiety Mediation Models

**FIGURE 2:**
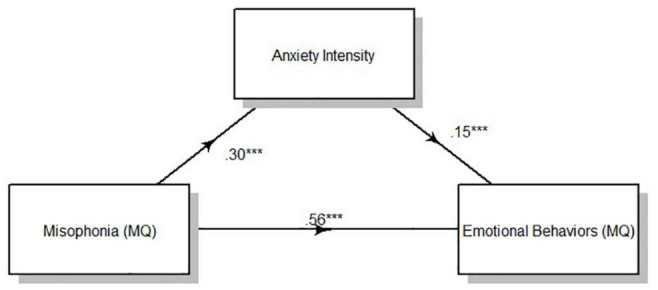
Overall mediation model for anxiety intensity. Standardized path coefficients. ***p < 0.001, all two tailed.

**FIGURE 3:**
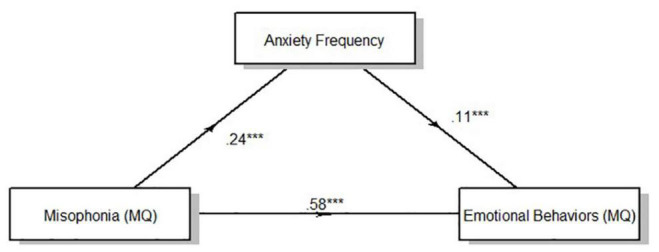
Overall mediation model for anxiety frequency. Standardized path coefficients. ***p < 0.001, all two tailed.

Result of the mediation analysis for anxiety intensity showed a direct effect of clinically relevant levels of misophonia determined from MQ severity scores on emotional behaviors indexed by the MQ. *F*_(1,330)_ = 106.52, *p* < 0.001. Anxiety intensity measured *via*self-report significantly mediated the effect of misophonia symptom severity on emotional behaviors to account for 7.41% of the total effect of MQ symptom severity on MQ emotional behaviors, *F*_(1,331)_ = 33.88, *p* < 0.001 (Figure 2). Anxiety frequency measured *via*self-report also significantly mediated the relationship between misophonia symptoms severity on emotional behaviors to account for 4.22% of the total effect of MQ symptom severity on MQ emotional behaviors, *F*_(1,331)_ = 20.52, *p* < 0.001 (Figure 3). The total unstandardized indirect effect of X (MQ symptom severity) on Y (MQ emotional behaviors) for the model with anxiety frequency was 0.07 and the total (direct) effect of X on Y for the model with anxiety intensity 0.13.

Results of the mediation analysis by cluster according to the three-cluster solution showed a direct effect of clinically relevant levels of misophonia determined from MQ severity scores on emotional behaviors (MQ) for all clusters [Cluster 1: *F*_(1,127)_ = 32.25, *p* < 0.001; Cluster 2: *F*_(1,90)_ = 30.99, *p* < 0.001; Cluster 3: *F*_(1,110)_ = 38.98, *p* < 0.001]. Anxiety intensity mediated the relationship between MQ severity scores and MQ emotional behaviors for cluster 1 and cluster 3, but not cluster 2 (Table 8). Anxiety intensity accounted for 18.96% of the total effect of MQ symptom severity on MQ emotional behaviors in cluster 1 and 9.87% of the total effect for cluster 3 but only 1.57% of the total effect for cluster 2. Anxiety frequency did not mediate the relationship between MQ severity scores and MQ emotional behaviors for any cluster. Figures 1, 2 show the overall mediation models for anxiety intensity and frequency, respectively, with the remaining mediation results reported in Table 8.

**TABLE 8:** Mediation model path coefficients.

	Standardized path coefficients		
	
Model path	Cluster 1	Cluster 2	Cluster 3
**Anxiety intensity model**			
Misophonia→Anxiety intensity	0.35***	0.12	0.29**
Anxiety intensity→Emotional behaviors	0.28**	0.06	0.17*
Misophonia→Emotional behaviors	0.41***	0.44***	0.46***
**Anxiety frequency model**			
Misophonia→Anxiety frequency	0.27*	0.08	0.18
Anxiety frequency→Emotional behaviors	0.15	–0.03	0.19*
Misophonia→Emotional behaviors	0.47***	0.45***	0.48***

*p < 0.05, **p < 0.01, ***p < 0.001, all two tailed. Mediation models for each cluster follow the same model structure as those in Figure 2 (anxiety intensity) and 3 (anxiety frequency).

### Diagnoses

Participants were asked to self-report current formal diagnoses that they held at the time of participation. It is important to note that participants were not required to submit medical records as proof of diagnosis; while the survey explicitly requested self-report of clinician-made formal diagnoses, some amount of self-diagnosis may contribute here. Self-reported diagnoses across the entire sample were evaluated by cluster membership based on the three-cluster solution (Table 9). All official diagnoses occurred with even frequency across all three clusters suggesting that clinically significant anxiety, depression, or personality disorder traits did not influence variable clustering. Anxiety disorders [i.e., General Anxiety Disorder (GAD), Obsessive-Compulsive Disorder (OCD), and Post-Traumatic Stress Disorder (PTSD)], and Depression were of specific interest and an additional analysis was conducted for diagnoses of interest by misophonia classification. All aforementioned anxiety disorders were combined into a single variable to test for group differences. Individuals that qualified for misophonia reported increased total anxiety diagnoses, OCD, PTSD, and GAD. Official diagnoses of depression, Panic Disorder, and Social Anxiety Disorder did not differ between misophonia groups (Table 10).

**TABLE 9:** Self-reported diagnoses by cluster membership.

	Percentage of participants		
	
Diagnosis	Cluster 1	Cluster 2	Cluster 3
**Eating disorders**			
Anorexia nervosa	2.2	1.5	4.2
Bulimia nervosa	2.2	0	0.8
**Neurodevelopmental disorders**			
Attention-deficit/hyperactivity disorder (ADHD)	10.7	6	5.9
Autism spectrum disorder (ASD)	0	0	0.8
Tic disorder	1.1	0	0.8
**Anxiety disorders**			
General anxiety disorder (GAD)	15.2	9.8	18.6
Obsessive compulsive disorder (OCD)	3.2	0.7	5.1
Panic disorder	0	1.5	0.8
Post-traumatic stress disorder (PTSD)	4.3	1.5	5.1
Social anxiety disorder	0	3	4.2
Anxiety disorders (total)	8.6	8.3	15.2
**Auditory disorders**			
Hearing loss	1.1	0.7	3.4
Hyperacusis	0	0	0
Selective mutism	0	0	0
Tinnitus	1.1	1.5	3.4
**Personality disorders**			
Obsessive compulsive personality disorder (OCPD)	1.1	0	0
Schizotypal personality disorder	0	0	0
**Depressive disorders**			
Depression	17.2	9.8	18.6
**Bipolar and related disorders**			
Bipolar disorder	0	0	1.7
**Other**			
Sensory processing disorder	0	0	0.8

*Categorization based on DSM-5 diagnostic categories.*

**TABLE 10:** Percent self-reported diagnoses by group.

	Group		
	
Diagnosis	No misophonia	Misophonia	Chi-squared
**Anxiety disorders**			
General anxiety disorder (GAD)	12.4	24	4.77*
Obsessive compulsive disorder (OCD)	1.3	11.1	14.16***
Panic disorder	0.6	1.8	0.78
Post-traumatic stress disorder (PTSD)	2	11.1	10.36**
Social anxiety disorder	3.1	0	1.71
Anxiety disorders (total)	7.6	27.7	17.81***
**Depressive disorders**			
Depression	13.4	22.2	2.67

Categorization based on DSM-5 diagnostic categories. *p < 0.05, **p < 0.01, ***p < 0.001.

Neurodevelopmental and sensory processing disorders were not frequently endorsed within the current sample with the exception of ADHD. Twenty-three individuals reported a formal diagnosis of ADHD and were included in the group difference analysis. No significant group differences were found between participants who qualified for misophonia and those that did not.

## Discussion

The current study aimed to replicate prior work evaluating misophonia in a large sample of university undergraduates with additional emphasis on symptoms of sensory hypersensitivity and symptom subgrouping. Our findings support a similar but slightly reduced misophonia prevalence rate within a non-clinical sample of undergraduate students, with approximately 15% of the current sample reporting clinically relevant levels of misophonia symptoms. Groupings were determined from MQ symptom severity scores identifying 54 participants who qualified for misophonia by exhibiting clinically relevant levels of misophonia symptoms. The relatively large percentage of participants who qualified for misophonia supports the conclusion that misophonia symptoms are common in non-clinical samples (Wu et al., 2014).

### Symptom Presentation

Our results support recent findings showing eating sounds and breathing/nasal sounds as the primary triggers for individuals with misophonia (Jager et al., 2020). People eating was the most frequently endorsed trigger item on the MQ trigger subscale, with approximately 45% of the sample reporting heightened sensitivity (i.e., selected *often sensitive* or *always sensitive*). S-Five trigger endorsement results supported MQ trigger subscale findings with increased reports of reduced tolerance for sounds related to eating. Though aversion to oral/nasal sounds is common, the frequency with which participants endorsed triggers unrelated to oral/nasal sounds is consistent with objective reports that individuals with misophonia find human non-oral/throat and non-human/nature sounds to be more aversive compared to individuals who do not have misophonia (Hansen et al., 2021).

Trigger endorsement rates for the MQ trigger items more frequently endorsed were relatively elevated compared to previous work (Edelstein et al., 2013; Wu et al., 2014). Trigger endorsement ranged from 20 to over 45% of the whole sample reporting they were either “often” or “always” sensitive to any given auditory trigger, apart from vowel and/or consonant sounds (∼ 9%). Participants who qualified for misophonia reported greater sensitivity to auditory stimuli classified under MQ trigger item categories compared to subclinical participants. Increased self-reported sensitivity to known misophonia triggers suggests participants who qualified for misophonia experience clinically relevant levels of auditory hypersensitivity across multiple stimuli.

S-Five triggers provided more detailed options for trigger endorsement and thus better-characterized responses from participants who qualified for misophonia. S-five sensory triggers covered a broad range of auditory stimuli that were more specific compared to MQ triggers (e.g., *crunching an apple* compared to *people eating*) with response options that better reflected commonly reported misophonia-specific reactions (e.g., annoyance, tolerance, aggressive behavior, and anxiety-induced avoidance). S-Five triggers also included non-auditory stimuli with differences between those without misophonia and individuals reporting clinical significant levels of misophonia symptoms on aversion to strong smells and some visual triggers suggesting pathways responsible for sensory hypersensitivities may be universally impaired in misophonia. A subset of participants endorsing the most extreme behavioral options on S-Five trigger items suggested a subpopulation with reduced tolerance and likely exhibition of extreme emotional or behavioral responses when exposed to specific stimuli (Vitoratou, 2018). Importantly, the S-Five indexes misophonia triggers in terms of emotional and behavioral responses to triggers resulting in a trigger section more specifically designed to depict the unique presentation of misophonia symptoms over general auditory hypersensitivity (Vitoratou, 2018; Vitoratou et al., 2021). Decreased tolerance for auditory triggers on the S-Five lends further support to the conclusion that the participants classified into the misophonia group by MQ symptom severity experienced increased sensory hypersensitivity to specific auditory stimuli with associated emotional/behavioral reactivity.

### Tinnitus and Sensory Processing Abnormalities

The incidence rate of tinnitus and misophonia co-occurrence was elevated suggesting that populations of individuals with misophonia have an increased risk for co-occurring sensory processing disorders. Screening questions regarding ear ringing have a high false positive rate when regarded alone, however increased severity of tinnitus symptoms on the THI in individuals with misophonia supports a likely true increase in co-occurrence which may be linked to basic sensory processing abnormalities relatively early in the auditory processing pathway. However, this interpretation may involve additional nuance as indicated by the cluster findings discussed below and by the functional limitations of the THI (Meikle et al., 2012).

### Cluster Results

Clustering our sample by symptom presentation provides a more nuanced approach to evaluating misophonia symptom characterization and understanding syndromic or spectrum representation in the disorder [Schroder et al., 2013; Erfanian et al., 2019; Jager et al., 2020; Swedo et al., 2021 (Preprint)]. The three-cluster solution identified a spectrum of symptom presentations ranging from no symptoms to severe symptom outcomes. These analyses identified a cluster (cluster 3) consisting of a severe neuropsychiatric symptom presentation with most participants exhibiting heightened broad-band sensory hypersensitivity, characteristics of ASD, and schizotypal personality characteristics. Cluster 3 also included individuals with the highest tinnitus severity scores, suggesting broad sensory involvement. Based on self-reported experiences of triggers in other sensory modalities, sensory hypersensitivities may be more specific to the auditory stimuli with moderately increased reports of difficulties in other sensory modalities for cluster 3. Cluster 1 consisted of the most severe presentations of misophonia symptoms, including increased reports of other sensory trigger experiences. Interestingly, cluster 1 contained more of the participants who qualified for misophonia compared to cluster 3, supporting the possibility that misophonia symptoms may represent a general risk pattern for more psychiatric disorder or even arise as an epiphenomenon of other disordered systems (e.g., subsyndromic ASD symptoms or tinnitus) in a relatively small subgroup of individuals with misophonia. Cluster 1 may represent a more “pure” form of misophonia that is less related to genetic risk for psychiatric disorder or specific sensory conditions like tinnitus and may respond differently to therapeutic intervention than more complicated forms with increased co-occurring psychiatric conditions. Regardless, participants clustered into cluster 1 still reported increased general sensitivity to sensory stimuli and more varied sensory experiences (i.e., other sensory triggers and severity of those trigger experiences) and exhibited increased characteristics of ASD compared to the relatively unaffected individuals in cluster 2. Given the differences in neuropsychiatric presentation across clusters, these subgroups may also reflect different underlying pathways related to difficulties in sensory processing (cluster 1) or higher-order cortical control (cluster 3), although this relationship remains to be experimentally validated. However, increased scores on the BAPQ across both clusters 1 and 3, particularly in behavioral rigidity symptoms, suggest individuals with misophonia show some overlap clinically with autism-like symptoms that may indicate similar underlying neural pathology (Hurley et al., 2007; Schroder et al., 2014).

The presence of multiple misophonia presentations suggests that misophonia symptoms may lie on a spectrum with varying levels of overlap with other brain disorders. The spectrum presentation conclusion is an important consideration for the approach to understanding and treating misophonia, previously assessed or diagnosed *via*questionnaire and recently *via*psychoacoustic methods [Schroder et al., 2013; Jager et al., 2020; Enzler et al., 2021; Hansen et al., 2021; Swedo et al., 2021 (Preprint)]. In many ways, misophonia shares a clinical presentation similar to the sensory and cognitive control aspects of ASD which could implicate similar potential underlying mechanisms for sensory sensitivity and emotional reactivity symptoms (Schroder et al., 2014; Jastreboff and Jastreboff, 2015; Daniels et al., 2020). Increased functional connectivity has been noted in relation to trigger sounds within individuals with misophonia, although top-down control of sensory systems has been less clearly addressed (Kumar et al., 2017). Increased and decreased functional connectivity, depending on the system, has also commonly been reported in ASD using multiple brain imaging technologies, with top-down control of sensory systems, cognition, and social skills particularly affected (Shou et al., 2017). If similar top-down control connectivity patterns can be established for misophonia, is possible that shared biological pathways primarily concerning the auditory system but potentially generalizable to other sensory systems could be implicated [Cerliani and Rouw, 2020 (Preprint)].

### Anxiety Mediation Models

One additional common co-occurring neuropsychiatric condition in misophonia is anxiety, which potentially amplifies the range of emotional reactivity observed in misophonia (Edelstein et al., 2013; Schröder et al., 2017; Cassiello-Robbins et al., 2021). Anxiety also potentially reflects a preemptive response to intolerable auditory stimuli (Edelstein et al., 2013; Wu et al., 2014). The anticipatory nature of anxiety symptoms typically noted in individuals with misophonia suggests a separate pathway from emotional processing pathways responsible for feelings of anger, panic, extreme irritation, and rage observed in response to trigger sounds (Edelstein et al., 2013). In the current study anxiety partially mediated the relationship between the severity of misophonia symptoms experienced and emotional behaviors based on MQ subscale scores and self-reported anxiety, similar to findings by Wu et al. (2014). Misophonia symptom severity was a positive predictor of emotional behavior scores, and increased symptom severity was predictive of increased emotional behaviors or reactions to trigger exposure. Both frequency and severity of anxiety symptoms mediated this relationship, however the effect of frequency was smaller relative to the effect of anxiety intensity. This relationship also differed by cluster, with anxiety severity only mediating relationships between misophonia symptoms and behaviors in clusters 1 and 3, where misophonia symptoms were most pronounced. When separated by cluster, the effect of anxiety frequency was no longer a significant mediator. A potential explanation for the reduced effect of anxiety frequency is thatparticipants exhibiting clinically relevant levels of misophonia may perceive themselves as living in a more perpetual state of anxiety rather than separable instances. Specifically, the anxiety experienced in relation to potential trigger exposure may follow patterns of volatility overprediction in autistic individuals. Autistic individuals tend to overlearn about the volatility of the changing environment leading to reduced surprise when events of change occur (Lawson et al., 2017). In other words, autistic individuals may experience sensory input overloads preventing accurate predictions *via*disruption of internal predictive models (i.e., bottom-up prediction errors that produce top-down predictions propagating downward causing failures to contextualize external sensory experiences) (van Boxtel and Lu, 2013; Gonzalez-Gadea et al., 2015). Auditory triggers potentially occur in all environments and individuals with increased misophonia symptom severity may predict the violation of their own sensory expectations at increased rates compared to those without misophonia (i.e., individuals with misophonia exist in a state of hyper-focus/selective attention for the possibility of trigger presence) (Lawson et al., 2017; Palumbo et al., 2018; Silva and Sanchez, 2019). Autistic individuals reportedly focus on details over holistic percepts following shifts in neurocognitive processing supporting meta-learning. Similar neural mechanisms underlying these features among individuals on the broad autism spectrum may be reflected in those experiencing misophonia (Gonzalez-Gadea et al., 2015; Smith et al., 2020; Todorova et al., 2021). Adjusted expectations about sensory experience potentially explains both symptom severity and behaviors in response to trigger exposure signifying one potential mechanism for sensory processing symptoms and symptoms of neuropsychiatric conditions. Additionally, the possibility of increased sensitivity to sounds reflecting reduced hearing thresholds or increased difficulty suppressing non-essential auditory information may contribute to anxiety intensity mediation of emotional behaviors over frequency of anxiety experiences, however this relationship has not been verified in the literature to date.

### Diagnoses

Prevalence rates of former DSM-IV Axis II disorders are known to be higher among individuals with misophonia relative to the general population. Participants were asked to self-report formal diagnoses from any of 21 specific DSM-5 diagnoses known to share symptoms with misophonia or co-occur with misophonia, particularly former DSM-IV Axis II diagnoses (Schroder et al., 2013). Formal diagnoses occurred with even frequency across all clusters suggesting that co-occurrence or symptom overlap of any one disorder does not contribute to the subcategorization of participants exhibiting clinically significant symptoms of misophonia. Rather, the increased scores on multiple neuropsychiatric subscales in Cluster 3 suggest a subgroup of individuals for whom misophonia symptoms may be driven by overall genetic or environmental factors contributing to psychiatric illness.

Regardless of cluster membership, individuals with misophonia exhibited elevated anxiety disorder diagnosis rates across the majority of diagnosis categories, similar to previous findings (Schroder et al., 2013; Jager et al., 2020).

Differences in formal diagnosis rates of general anxiety disorder between groups further support the conclusion that anxiety was not an exposure-response to auditory triggers in individuals with misophonia but reflected a preemptive anxiety response to potential trigger exposure (Lawson et al., 2017; Jager et al., 2020). Increased rates of OCD coupled with elevated behavioral rigidity scores across both clusters 1 and 3 further suggest potential obsessive preoccupations with auditory triggers that reflect preemptive responses to auditory trigger exposure. Finally, increased formal diagnoses of PTSD among those with misophonia is a relatively unique finding, albeit one that must be interpreted in light of the small number of PTSD cases in this sample. By percentage, the number of individuals with misophonia that reported a formal PTSD diagnosis (∼20%) matches the recent findings of Cassiello-Robbins et al. (2021). Increased PTSD is consistent with increased anxiety in misophonia and may represent a specific subsample where misophonia symptoms are tied to uniquely traumatic experiences (Jager et al., 2020; Cassiello-Robbins et al., 2021). Further, previous reports suggest that individuals with misophonia exhibit increased clinician-rated symptoms of personality disorders linked to increasing symptom severity, but not other conditions. Following conclusions made by Cassiello-Robbins et al. (2021), the range of psychiatric symptoms associated with misophonia may uniquely reflect mechanisms of misophonia over other discrete psychiatric conditions.

### Limitations

The current study was an effort to explore misophonia symptoms of sensory hypersensitivity using clinical measures and participants from a young adult, non-clinical sample. The major limitation for interpretation was the lack of psychiatric evaluation of participants to address the background of misophonia symptoms, anxiety, and potential similarities between misophonia and ASD. Formal diagnoses were by self-report and limited in number, thus limiting interpretation of their impact on misophonia symptoms beyond that of more general variation in neuropsychiatric symptoms as measured from the survey scales (e.g., BAPQ, SCQ). Survey items regarding anxiety frequency and intensity were broad and encompassed any form of anxiety, also limiting the interpretations on the role of specific forms of anxiety in misophonia. A minor limitation in tinnitus evaluation was the self-reported nature of whether they experience tinnitus (i.e., ringing in the ears). As a result, some reported tinnitus may reflect experiences of typical aural fluctuations. Use of the THI poses minor limitations in functional use and should be interpreted as an index of tinnitus impact on quality of life (Meikle et al., 2012).

Use of an older version of the S-Five (Vitoratou, 2018) for the current study reflects a minor study limitation. The newest version was published after the formation and during the administration of the survey. Future research may take advantage of the newly described S-Five trigger endorsement factor structure to compare to MQ symptoms severity scores for misophonia group determination outcomes (Vitoratou et al., 2021). Lastly, other self-report measures exist for evaluating misophonia not used in the current study that may prove useful. The MisoQuest survey was developed based on the diagnostic criteria set forth by Schroder et al. (2013) and may bridge the gap between physical evaluations and the use of surveys to identify misophonia (Siepsiak et al., 2020). Survey measures used for the current evaluation of misophonia were selected based on prevalence of use in the literature and, in the case of the MQ, to replicate previous findings (Wu et al., 2014).

## Conclusion

This study found two potential subgroups of individuals with misophonia: one subgroup with more “pure form” misophonia represented by the most severe misophonia symptoms but relatively few co-occurring conditions, and one subgroup with an increased number of co-occurring conditions which may represent misophonia as an epiphenomenon of increased risk for neuropsychiatric conditions in general. Subgroups of individuals with misophonia who may represent differential neuropsychiatric risk patterns and thus potentially different causative factors creates new demand for exploring the relationship between misophonia sensory symptoms, misophonia emotional reactivity/behavioral symptoms, and related neuropsychiatric conditions such as ASD and anxiety with an emphasis on neural mechanisms. Future work should evaluate auditory stimuli and responses to complex auditory stimuli (e.g., speech), as well as specific experimental assessment of cognitive control difficulties in individuals with misophonia to address the potential syndromic or subgrouped relationship between misophonia, sensory processing disorders, executive function, and state/trait levels of anxiety (Jochaut et al., 2015).

## Data Availability Statement

The raw data supporting the conclusions of this article will be made available by the authors, without undue reservation.

## Ethics Statement

The studies involving human participants were reviewed and approved by the University of Oklahoma Institutional Review Board. The patients/participants provided their written informed consent to participate in this study.

## Author Contributions

JN aided in study design, analyzed the data, and wrote the manuscript. SK aided in critical clinical interpretation of results and discussion, and contributed to the manuscript. DN designed the spectrum survey and aided in initial study design. LE conceived of the project, oversaw study design, data analyses, and manuscript preparation. All authors contributed to the article and approved the submitted version.

## Conflict of Interest

The authors declare that the research was conducted in the absence of any commercial or financial relationships that could be construed as a potential conflict of interest.

## Publisher’s Note

All claims expressed in this article are solely those of the authors and do not necessarily represent those of their affiliated organizations, or those of the publisher, the editors and the reviewers. Any product that may be evaluated in this article, or claim that may be made by its manufacturer, is not guaranteed or endorsed by the publisher.
